# The Effect of Using a Fetal Pillow in Second-Stage Cesarean Sections on Fetal and Maternal Outcomes in Comparison to Other Usual Methods: A Retrospective Study

**DOI:** 10.7759/cureus.82158

**Published:** 2025-04-12

**Authors:** Amr A Lila, Ramy Noureldeen, Clare Redfearn

**Affiliations:** 1 Obstetrics and Gynecology, East Kent Hospitals University NHS Foundation Trust, Margate, GBR

**Keywords:** disimpaction, fetal outcome, fetal pillow, maternal outcome, second-stage cesarean section

## Abstract

Objective

To evaluate the impact of using the Fetal Pillow on maternal and fetal outcomes during second-stage cesarean sections compared to conventional methods, including vaginal disimpaction, abdominal disimpaction, breech extraction, wound extension, and the use of tocolytics.

Methodology

This retrospective cohort study analyzed 100 second-stage cesarean sections performed at Queen Elizabeth The Queen Mother Hospital (QEQM) and William Harvey Hospital (WHH) between March 2022 and June 2024. The Fetal Pillow was used in 29 cases, while conventional methods were employed in 71 cases. Maternal outcomes assessed included estimated blood loss (EBL), length of hospital stay, and uterine extensions. Fetal outcomes included Special Care Baby Unit (SCBU) admission, arterial cord pH, and fetal injuries.

Results

The use of the Fetal Pillow did not result in statistically significant differences in maternal outcomes, including EBL (*P* = 0.418), hospital stay (*P* = 0.703), and uterine extensions (*P* = 0.554). Similarly, there were no significant differences in SCBU admissions (*P* = 0.578) or arterial cord pH < 7.05 (*P* = 0.432). However, cases where the Fetal Pillow was used showed a significant increase in fetal injuries such as facial bruises (*P* = 0.005). Notably, the median EBL and hospital stay were lower in the Fetal Pillow group, although these differences were not statistically significant.

Conclusions

The use of the Fetal Pillow in second-stage cesarean sections did not result in statistically significant differences in most fetal or maternal outcomes when compared to the conventional methods. However, cases where the Fetal Pillow was utilized demonstrated a lower median EBL and a shorter median hospital stay. The observed increase in fetal injuries associated with the Fetal Pillow seems to be linked to its use after unsuccessful instrumental deliveries. Future research should focus on identifying specific clinical scenarios where the Fetal Pillow can offer maximal benefit while minimizing risks.

## Introduction

Cesarean section (CS) is one of the most commonly performed obstetric procedures worldwide, with rates continuing to rise in both developed and developing countries. Approximately 5% of all CSs occur at full cervical dilatation, often requiring additional maneuvers to facilitate delivery due to the deeply engaged fetal head. This condition, known as impacted fetal head (IFH), poses significant risks to both the mother and neonate, including uterine incision extensions, excessive maternal bleeding, fetal trauma, and increased operative time [1].

The second stage of labor is defined by full cervical dilation and ends with the delivery of the baby. When a CA is performed at this stage, the fetal head is often deeply impacted in the maternal pelvis, making its extraction technically challenging. Second-stage CSs account for up to 6% of all CSs, with their incidence increasing due to rising primary cesarean rates and declining proficiency in assisted vaginal deliveries [1,2]. The growing preference for CSs over instrumental deliveries has led to an increased number of second-stage procedures, posing new challenges for obstetricians [3].

Various techniques have been developed to manage IFH, including manual vaginal disimpaction, reverse breech extraction, and the use of cephalic elevation devices such as the Fetal Pillow. The Fetal Pillow is an inflatable device designed to elevate the fetal head before uterine incision, aiming to facilitate safer and easier delivery. Although some early studies suggested that the Fetal Pillow might reduce maternal morbidity by minimizing uterine extensions and blood loss, more recent data have produced conflicting results. Notably, large retrospective studies have shown no significant reductions in these outcomes, and concerns remain regarding its effectiveness when used following unsuccessful instrumental deliveries [4,5].

Second-stage CSs are associated with higher maternal and neonatal complications, including increased rates of postpartum hemorrhage and risks in subsequent pregnancies, such as uterine rupture, as noted in the literature on impacted fetal head delivery [3,4,6]. The choice of fetal head disimpaction technique plays a crucial role in mitigating these risks. While the push and pull methods (including the reverse breech extraction) have been traditionally used, cephalic elevation devices such as the Fetal Pillow have been introduced as a potential alternative to facilitate atraumatic delivery [6].

Despite its increasing adoption, the optimal role of the Fetal Pillow in second-stage CSs remains unclear. Some studies advocate its use as a method to decrease maternal complications, whereas others raise concerns about increased neonatal trauma when used after failed instrumental deliveries [5]. Given the lack of consensus and limited robust data, further research is needed to evaluate the risks and benefits associated with its application.

This study aims to evaluate the impact of the Fetal Pillow on key maternal outcomes, including estimated blood loss (EBL), hospital stay, and uterine extensions, as well as fetal outcomes such as Special Care Baby Unit (SCBU) admissions, arterial cord pH, and neonatal injuries. By comparing these outcomes with those observed using conventional disimpaction methods, this research seeks to determine whether the Fetal Pillow offers a significant clinical advantage and identify areas requiring further investigation, an approach also examined in previous studies such as by Hanley et al. [7].

## Materials and methods

Study design and setting

This retrospective cohort study was conducted at Queen Elizabeth The Queen Mother (QEQM) Hospital and William Harvey Hospital (WHH) between March 24, 2022, and June 6, 2024. The study aimed to evaluate the effect of the Fetal Pillow on maternal and fetal outcomes in second-stage cesarean sections compared to conventional disimpaction methods.

Study population

Inclusion Criteria

Women undergoing second-stage cesarean section at QEQM and WHH during the study period. Cases where fetal head disimpaction was attempted using either the Fetal Pillow or conventional techniques. Complete medical records available for maternal and fetal outcomes.

Exclusion Criteria

Cases with incomplete documentation regarding disimpaction techniques and maternal/fetal outcomes. Cesarean sections performed at other stages of labor (e.g., first-stage cesarean deliveries). Women with pre-existing maternal conditions significantly affecting cesarean outcomes (e.g., severe coagulation disorders).

Sampling technique

A consecutive sampling method was used to identify eligible cases. All second-stage cesarean sections performed within the study period that met the inclusion criteria were included in the analysis.

Data Collection

Data were retrospectively extracted from electronic medical records, operative notes, and discharge summaries. The following variables were recorded:

Maternal outcomes: Maternal outcomes included EBL, measured in milliliters, and hospital stay duration, recorded in days. Uterine extensions, such as J-incisions, T-incisions, or other documented types, were also evaluated.

Fetal outcomes: Fetal outcomes included admission to the SCBU, arterial cord pH values below 7.05 (indicating potential acidosis), and documented fetal injuries, such as facial bruises, scalp lacerations, or forceps marks.

Cases were stratified based on the method used for fetal head disimpaction into two groups: the Fetal Pillow group and the Conventional Methods group, which included abdominal disimpaction, vaginal disimpaction, breech extraction, and wound extension.

Statistical analysis

Data were analyzed using IBM SPSS Statistics version 26 (IBM Corp., Armonk, NY). Statistical tests applied included the Chi-square test for categorical variables (e.g., uterine extensions, fetal injuries, SCBU admissions), and the independent t-test for continuous variables (e.g., estimated blood loss and hospital stay duration). One-way ANOVA was used for group comparisons when appropriate. A *P*-value of < 0.05 was considered statistically significant, while a *P*-value of < 0.001 was regarded as highly statistically significant.

Ethical considerations

This was a retrospective study utilizing data collected from hospital records. As there was no direct patient involvement or participation of human subjects, ethical approval was not required.

## Results

General findings

There was no statistically significant difference in body mass index (BMI) between patients in whom the Fetal Pillow was used and those managed with other disimpaction methods. Normal BMI accounted for 42% of cases, overweight for 23%, and obesity (including morbid obesity) for 34%.

The primary reasons for second-stage cesarean sections included prolonged second stage (40%), failed trial of labor (32%), and fetal hypoxia (19%). Other reasons, such as malpresentation and maternal request, were less common.

Additional maneuvers were sometimes used in conjunction with the Fetal Pillow, including vaginal disimpaction and tocolysis, reflecting the complexity of cases requiring cesarean delivery at this stage.

Labor events, such as fetal head position and attempts at instrumental delivery, often influenced the choice of disimpaction method. Occipitoposterior and occipitotransverse fetal positions were predominant in these cases, accounting for 71% of the total.

Maternal outcomes

The mean EBL was lower in the Fetal Pillow group (721.76 ± 326.11 mL) compared to the conventional methods group (855.44 ± 500.16 mL). However, this difference was not statistically significant (*P* = 0.418). Table 1 summarizes the comparison of EBL between different methods, showing no significant difference between groups.

**Table 1:** Comparison of maternal outcomes between cases where the Fetal Pillow was used and those managed with conventional methods (n = 100). The comparison of EBL between different methods showed no significant difference (*P* = 0.418). The comparison of hospital stays between different methods also showed no significant difference (*P* = 0.703), and the comparison of uterine extensions showed no significant difference (*P* = 0.554).

Study group (*n *= 100)			Fetal Pillow used			Test	*P*-value
			Yes (*n *= 29)		Another maneuver (*n *= 71)	*X*^2^/*Z*	
EBL median (IQR)			616 (504.5-868.75)		700 (497.5-1200)	-0.810	0.418
Hospital stays (Days post-partum), Median (IQR)			1 (1-2)		2 (1-3)	-0.381	0.703
Wound extensions	Yes	n	10	29		0.350 (*X*^2^)	0.554
		%	25.6%	74.4%			

The length of hospital stay was similar between groups: 2.52 ± 2.57 days for the Fetal Pillow group and 1.96 ± 1.12 days for conventional methods (*P* = 0.703). Table 1 also presents the comparison of hospital stays, which showed no significant difference.

Uterine extensions, including J-incisions and T-incisions, occurred in 10% of cases where the Fetal Pillow was used and 29% in the conventional methods group. This difference was not statistically significant (*P* = 0.554). As seen in Table 1, the comparison of uterine extensions across groups did not show a statistically significant difference.

Fetal outcomes

SCBU admissions were comparable between groups, with no statistically significant difference (*P *= 0.578). Table 2 presents a comparison of SCBU admissions between the Fetal Pillow and conventional methods groups, indicating no statistically significant difference.

**Table 2:** Comparison of fetal outcomes between cases where the Fetal Pillow was used and those where it was not (n = 100). There was no significant effect on admission to SCBU (*P* = 0.578). Similarly, there was no significant effect on arterial cord blood pH < 7.05 (*P* = 0.432). However, there was a significant effect on fetal injuries, such as facial bruises, scalp lacerations, and forceps marks, with a *P*-value of 0.005.

Study group (*n *= 100)			Fetal Pillow used		Test	*P*-value
			Yes (*n *= 29)	Another maneuver (*n *= 71)	*X*^2^	
Fetal injury	Face bruises	n	11	6	14.696	0.005*
		%	64.7%	35.3%		
	Forceps marks	n	1	1		
		%	50.0%	50.0%		
	Scalp lacerations	n	1	2		
		%	33.3%	66.7%		
Arterial pH < 7.05	Yes	n	2	2	1.677	0.432
		%	50.0%	50.0%		
Admission to SCBU	Yes	n	2	3	0.309	0.578
		%	40.0%	60.0%		

Arterial cord pH values <7.05 showed no significant difference between the Fetal Pillow and conventional methods groups (*P* = 0.432). As shown in Table 2, arterial cord blood pH values did not significantly differ between groups.

Fetal injuries were significantly more common in the Fetal Pillow group (37.9%) compared to the conventional methods group (16.2%). Injuries included facial bruises, scalp lacerations, and forceps marks, with a statistically significant *P*-value of 0.005. Table 2 highlights the significant difference in fetal injuries, with a higher prevalence observed in the Fetal Pillow group compared to conventional methods.

Additional observations

The Fetal Pillow was often used following unsuccessful instrumental deliveries, which may account for the observed increase in fetal injuries.

Among the 29 cases where the Fetal Pillow was used, 14 required additional maneuvers to deliver the baby, underscoring the complexity of these cases and raising concerns about the efficacy of the Fetal Pillow.

There was no significant difference in the outcomes based on BMI, suggesting that patient weight did not influence the effectiveness of the Fetal Pillow.

## Discussion

Key findings

This study highlights that the Fetal Pillow, while potentially reducing maternal complications such as EBL and uterine extensions, did not achieve statistically significant improvements in most maternal or fetal outcomes. Notably, the use of the Fetal Pillow in our cohort was associated with a significant increase in fetal injuries, such as facial bruises and scalp lacerations, compared to conventional methods, primarily due to its use after unsuccessful instrumental deliveries, a trend also noted in previous studies [4,5,8].

Additionally, nearly half of the cases requiring the Fetal Pillow in our study involved additional maneuvers, reflecting the complex clinical scenarios in which it was employed and raising concerns about its efficacy. These findings align with research by Sadler et al. [4] and Jones et al. [8], who reported that second-stage cesarean sections involving cephalic elevation devices frequently necessitate additional interventions, increasing the risk of fetal trauma.

Interpretation of results

The observed increase in fetal injuries may stem from its use following failed instrumental deliveries, where the fetal head is already subject to increased manipulation. This aligns with previous findings, emphasizing the risks of combined disimpaction techniques in complex second-stage cesarean sections [4]. Moreover, excessive fetal manipulation during second-stage cesarean sections may exacerbate fetal morbidity, particularly in cases where multiple extraction methods are attempted sequentially, as observed in our cohort. This concern is also reflected in the literature on impacted fetal head delivery [3,4].

Despite these concerns, the lower mean EBL and similar hospital stay durations observed in our study suggest that the Fetal Pillow may still offer maternal benefits in certain scenarios, a possibility also noted by Sacre et al. [5]. A recent cohort study by Hanley et al. suggested that techniques reducing traction forces on the fetal head could minimize maternal trauma while maintaining fetal safety, a finding that warrants further investigation in larger cohort studies [7].

Comparison with existing literature

Previous studies have demonstrated that the method of fetal extraction plays a crucial role in maternal and neonatal outcomes. Peled et al. [3] found that different extraction techniques in second-stage cesarean delivery had varying impacts on preterm birth and uterine incision extensions. The reverse breech extraction method, for instance, has been associated with lower rates of complications compared to the push method, emphasizing the need to consider alternative disimpaction strategies [1]. A review by van der Krogt et al. [1], along with findings from Jones et al. [8], indicated that cephalic elevation devices, while beneficial, must be used cautiously to avoid excessive fetal head compression

The study by Gurashi et al. [6] highlighted the increased maternal complications associated with second-stage cesarean sections, including postpartum hemorrhage, uterine extensions, and prolonged hospital stays. These observations are consistent with our cohort, which focused on second-stage procedures. However, our findings align with those of Sacre et al. [5], who reported no significant difference in maternal outcomes between conventional disimpaction methods and the use of the Fetal Pillow. Similarly, a retrospective study by Vashi et al. [2] reported no significant difference in overall maternal morbidity between the different methods, further supporting the need for additional randomized trials to clarify its role in clinical practice, as echoed in other clinical and simulation-based studies [8,9].

Additionally, recent research has suggested that training and standardized protocols for managing impacted fetal head scenarios may improve clinical outcomes and reduce complications [6,10]. Simulation-based training has been shown to enhance obstetricians’ technical performance and confidence when managing difficult second-stage cesarean sections [10,11]. A randomized study by van der Scheer et al. [10] demonstrated that structured training improved neonatal outcomes by reducing the rate of failed disimpaction attempts. Incorporating standardized training programs for Fetal Pillow use may help optimize its application and minimize associated risks, as highlighted in clinical outcome studies [5].

Future directions and clinical implications

Future research should aim to conduct prospective randomized controlled trials comparing the Fetal Pillow to alternative extraction techniques [9,12], evaluate long-term neonatal outcomes associated with fetal head elevation devices [5], and assess the impact of operator experience and training on clinical outcomes [13]. A multicenter trial investigating standardized protocols for managing impacted fetal head deliveries would be invaluable in guiding future obstetric practice [14].

## Conclusions

This study demonstrates that the Fetal Pillow, while showing potential maternal benefits such as reduced estimated blood loss and shorter hospital stays, does not consistently achieve statistically significant improvements in maternal or fetal outcomes compared to conventional methods.

The findings suggest that the Fetal Pillow may be more effective when employed in carefully selected clinical scenarios, and its routine use should be guided by further evidence. Future studies should aim to identify optimal usage protocols and evaluate their effectiveness in larger, prospective cohorts. Enhanced training and clear guidelines are essential to minimize risks and maximize its potential benefits in managing second-stage cesarean sections.
